# Frequency of Stillbirths in a Tertiary Care Hospital, Karachi

**DOI:** 10.12669/pjms.321.8558

**Published:** 2016

**Authors:** Muhammad Ayaz Mustufa, Shazia Kulsoom, Ifra Sameen, Khemchand N Moorani, Ashfaqe Ahmed Memon, Razia Korejo

**Affiliations:** 1 Muhammad Ayaz Mustufa, MBE, Ph.D, Senior Research Officer, Pakistan Medical Research Centre, National Institute of Child Health, Karachi, Pakistan; 2 Shazia Kulsoom, MBBS, FCPS, Assistant Professor, Department of Pediatric Medicine Unit III, National Institute of Child Health, Karachi, Pakistan; 3 Ifra Sameen, MBBS, DCH, MCPS, FCPS, Assistant Professor, Department of Pediatric Medicine Unit III, National Institute of Child Health, Karachi, Pakistan; 4 Khemchand N Moorani, MBBS, MCPS, FCPS, Professor of Pediatrics, Department of Pediatric Medicine Unit III, National Institute of Child Health, Karachi, Pakistan; 5 Ashfaqe Ahmed Memon, B.SC Biostatics, Statistical Officer, Pakistan Medical Research Centre, National Institute of Child Health, Karachi, Pakistan; 6 Razia Korejo, Professor of Obs & Gyne, Bahria University of Medical & Dental College, Karachi, Pakistan

**Keywords:** Still births, Primiparous mothers, Gestational age

## Abstract

**Background and Objective:**

Pakistan accounts for the highest stillbirth rate in the world. Therefore, this observational study was planned to determine the prevalence of stillbirths and its associated demographic characteristics in the given context. Hence our objective included: To determine the frequency of stillbirths with reference to parity and gestational age in a tertiary care public hospital, Karachi. To determine the socio-demographic characteristics of families with stillbirths.

**Methods:**

All pregnant mothers who delivered stillbirth babies at Gynaecology and Obstetrics ward of Jinnah Postgraduate Medical Center, Karachi a tertiary care facility were prospectively enrolled from October 2012 to September 2013. Deliveries occurred before 28 weeks of gestational age were excluded. Gestational age was confirmed from hospital record and attending physicians. Data was collected on predesigned proforma and analyzed using descriptive statistics.

**Results:**

Among 7708 registered deliveries, 137 were stillbirths. A total of 84 mothers were primiparous and 12% of mothers were below 20 years at the time of delivery. Majority of stillbirths were macerated type (80.3%) and 20% were fresh stillbirth. About 55% of still births occurred between 33-37 weeks and 20% between 28-32 weeks. Almost 80% (109) of stillbirths were low birth weight and only 20% (28) were normal birth weight.

**Conclusion:**

This study shows that stillbirths are more common in primiparous mothers in a given context. Conducting awareness sessions with special focus on antenatal and obstetrical care of primiparous may be helpful to reduce still births.

## INTRODUCTION

Globally more than four million babies die in the neonatal period and there is a marginal improvement in neonatal survival during last two decades.^1^ WHO has reported stillbirth as fifth leading cause of death worldwide and represent a commonest preventable devastating pregnancy outcome.^2^ Stillbirths are high in developing world and there is a very little decline in stillbirth in the developed countries.^1^-^4^ In developed countries, most of the stillbirths are antepartum deaths(>40 %) due to placental dysfunction, growth restriction and many other unknown causes.^3^,^5^

Maternal obesity and smoking are considered as a highly modifiable risk factors and primiparity as non-modifiable risk factor.^5^-^7^ In high-income countries, the risk of stillbirths in primiparous older mothers (>35 years) is higher than young primiparous(< 35 years) due to delay in childbearing.^1^,^3^,^5^,^7^

The prevalence of stillbirth rates vary in different studies based on location, health care facilities and socioeconomic settings. Stillbirth rate in most of the developed countries are less than 5 per 1000 births whereas it ranges from 20 - 40 per 1000 births in the developing countries.^6^-^8^ Stillbirth rate of low-middle income countries (LMIC) is ten times higher than developed world.^9^ Worldwide, 2.7 million stillbirths occur each year (7,200 /day), two-third of these occur in South Asia and sub-Saharan Africa and 55% stillbirths are occurring in rural communities including Pakistan.^3^,^8^,^10^-^12^

Globally, among 4 million neonatal deaths that occur every year, 98% deaths occur in the resource poor countries.^3^,^10^-^12^ Though, these estimates are alarming and shows that neonatal deaths and stillbirths are almost equal but stillbirths have never been given due importance as global priority in millennium developmental goals.^1^,^3^,^5^

In Pakistan, reported stillbirth rate ranges from 36 - 98 per 1000 birth.^8^,^10^,^11^ Five major risk factors of stillbirth are maternal infections in pregnancy, maternal disorders (pre-eclampsia and diabetes), child birth complications, foetal growth restriction and congenital abnormalities.^1^,^5^-^9^,^12^,^13^

In Pakistan, nearly two-third of deliveries continue to take place at home and more than 62% of all deliveries are assisted by untrained birth attendants.^14^,^15^ Most of the research has been hospital-based with the prime focus on maternal and neonatal deaths rather than stillbirths.^16^ Furthermore, more than two fold rise in hospital based deliveries is reported in the last decade highlighting the provision of antenatal and obstetrical care.^17^-^19^

Therefore, considering the above facts and inclining trends of facility based deliveries, we conducted this study with the objectives; to determine the frequency of stillbirths with reference to parity and gestational age in mothers and to determine the socio-demographic status associated with stillbirths.

## METHODS

The data collection process was completed in a one year period. Informed written consent was taken from all pregnant mothers after explaining purpose of the study. The participants were given the right to disassociate from the study at any time. Ethical clearance was obtained from institutional ethical review committee (IERC) to conduct the study.

This descriptive case series study consisted of 137 stillbirths after taking consent from mothers delivered in the department of Obstetrics and Gynaecology, Jinnah Postgraduate Medical Centre (JPMC), Karachi from October, 2012 to September, 2013. All mothers with minimum of three antenatal visits and attended by duty doctor were studied.

In this study, stillbirth was defined as foetal death which occured at or after 28 weeks of gestation and without signs of life (breathing, crying, heart beat and movement).^2^ Further categorization was done as macerated type when signs of maceration (skin discoloration, sloughing of skin and overriding of sutures) were present and fresh still births (intrapartum) when no signs of maceration were present.^2^,^9^ Based on gestational age stillbirths were categorized as term (> 37 weeks), late preterm(> 32-37 weeks) and early preterm(>28- 32 weeks). Birth weights of stillbirths were catergorized as per standards as normal birth weight (NBW > 2.5 kg), LBW (<2.5-1.5kg), very LBW (<1.5-1 kg)) and very very LBW(<1kg). In this study, mothers were categorized into three age groups; age less than 20 years, 20 to 35 years and more than 35 years. Similarly, we classified maternal parity as primiparous (first pregnancy), biparous (1-2) and multiparity(≥3).

Semi structured proforma was used to collect socio-demographic characteristics of the study population including parental education level, family occupation and income. In second part, maternal age, parity, gestational age and pregnancy outcome were recorded. Information regarding gestational age was collected from mothers’ file. Deliveries which occurred before 28 weeks of pregnancy were excluded. Descriptive statistical tools were used for data analysis of still births and other variables.

## RESULTS

Among 7708 registered deliveries, 137 were stillbirths. The socio-demographic characteristics of 137 families who had stillbirths are shown in Table-I. There was no gender difference in stillbirths with male (51%) to female (49%) ratio is almost same. In our study, majority of stillbirths belonged to Urdu speaking families (50.4%) followed by Sindhi’s (20%). Around 58% of families had their monthly earnings less than 10,000 Pak rupees (Approx. 100 USD) and in more than 50% of families, the source of earning was daily wage or labour. More than 40% of mothers and 39% of fathers of the study population did not receive formal education at all.

**Table-I:** Sociodemographic characteristics associated with stillbirths (n=137).

*Characteristics*	*Mothers N (%)*	*Fathers N (%)*

*Parental Education*		
No formal education	55 (40.14)	32 (23.36)
Primary	54 (39.42)	48 (35.03)
Secondary	23 (16.8)	34 (24.82)
College	5 (3.6)	23 (16.79)

*Family Income*	*Number*	*Percentage*

Low (<5000)	5	3.6
Middle (5000 - 10000)	74	54
Upper (>10000)	58	42.3

*Ethnicity*	*Number*	*Percentage*

Urdu	69	50.4
Sindhi	27	19.7
Punjabi	16	11.7
Pashto	8	5.8
Balouchi	8	5.8
Others	9	6.6

*Family head occupation*	*Number*	*Percentage*

Labour	64	46.72
Govt/Private Job	54	39.41
Small Business	14	10.21
Technical Worker	5	3.65

Among 137 stillbirths, 84 (61%) mothers were primiparous, 22(16%) were multiparous and 31(23%) were biparous. (Table-II) About 101(74.72%) of mothers were between 20-35 years age, 20(15%) mothers were above 35 years and 16(11.68%) were less than 20 years.

**Table-II:** Maternal characteristics associated with Stillbirths (n=137).

Maternal Age	Number	Percentage
< 20	16	11.68
20 - 35	101	73.72
>35	20	14.60
Gravity		
Primiparous	84	61
Biparous	22	16
Multiparous	31	23

Fetal characteristics associated with stillbirths are shown in Table-III. Majority of stillbirths (80%) were of macerated type whereas remaining (20%) were fresh stillbirths. Stillbirths were late preterm in 55.5%, early preterm in 33.6% and only 11% were term. Among 137 stillbirths, around 80% were low birth weight and only 20% were normal birth weight (Table IV).

**Table-III:** Fetal characteristics associated with stillbirths (n=137).

*Characteristics*	*Number*	*Percentage*
*Gender*		
Male	70	51
Female	67	49

*Gestational age*	*Number*	*Percentage*

Early Preterm (>28-32 weeks)	46	33.58
Late preterm (>32-37 weeks)	76	55.47
Term (>37 weeks)	15	10.95

*Type of stillbirths*	*Number*	*Percentage*
Macerated	110	80
Non-macerated	27	20

**Table IV:** Birth weight categories of 137 stillbirths.

Birth weight (BW)	N (%)	Mean±SD	Range
Normal BW (>2.5 kg)	28(20.44)	2.89+0.25	2.5-3.3
Low BW (<2.5-1.5 kg)	42(30.66)	1.87+0.34	1.5-2.4
Very LBW (<1.5 -1 kg)	37(27.00)	1.16+0.14	1.0-1.4
Very very LBW (< 1kg)	30(21.90)	0.52+ 0.25	0.1-0.9

## DISCUSSION

Pakistan has highest stillbirth rate in the world, which is an alarming scenario.^19^ Current study revealed a high estimate of stillbirth i-e 18 per 1000 births, which is relatively lower than published data from Pakistan.^6^,^18^ Possible reasons may be tertiary care study setting and enrollment of booked cases only. Meanwhile, it is not worthy that around three fold rise in hospital based deliveries in last two decades highlight the provision of antenatal and obstetrical care.^17^,^20^ Higher stillbirth rates have been reported from developing countries including Nepal, India and Pakistan.^8^,^13^,^15^,^21^-^23^

In a most recent population based multicountry study over a period of three years from seven countries by Global Network showed a stillbirth rate from Pakistan as 56.5 per 1000.^18^ The reason of high stillbirth rate might be a rural stting whereas our study was from tertiary care setting of cosmopolitian city. Therefore, single stting based findings cannot be generalized.

We observed that majority of mothers (73.22%) who delivered stillbirths were between 20-34 years of age. Similar findings have been reported in other studies from Pakistan, India and Nepal.^8^,^11^,^14^,^19^,^23^ In contrast stillbirths have been reported among old aged (>35 years) mothers from developed countries.^1^,^7^,^9^

Primiparous mothers are at increased risk of stillbirth.^5^-^8^,^14^,^17^ Current study showed a high proportion of stillbirths in primiparous mothers (61%). Though early detection of risk factors (maternal or foetal) in primiparous mothers may decrease the stillbirth rate but due to lack of optimal antenatal care, primiparous mothers are more prone to stillbirth deliveries in our study.

In comparison, low risk of stillbirth in primiparous mothers has been reported from developed countries which may be due to well established infrastructure.^1^,^5^,^7^,^24^

We found that 76% stillbirths were preterm and among them 55.5% were late preterm and only 10% were term stillbirths. Our findings are similar to other studies from developing and developed countries.^1^,^5^,^14^,^18^

Surprisingly, our findings showed a high rate of macerated stillbirth (80%) which is much higher than previously published work from Pakistan.^8^,^18^,^21^ A wide range of macerated stillbiths rate (3.6-45.8%) has been reported from developing countries.^8^,^18^,^22^ High rate of macerated stillbirth in our study may be attributed to multiple factors like late arrival of mothers which may be due to late referal by health personels, long distance, lack of transport and lack of awareness.

Stillbirths with sign of maceration suggest foetal death at least 12 hours prior to delivery and those without signs of maceration are intrapartum deaths. This categorization emphasises that improved antenatal care reduces the risk of macerated stillbirth while quality care during labour and delivery may decrease the fresh stillbirth.^1^,^4^

Considering the sociodemographics, poverty is the prime and prevalent factor limiting the access to care, leading to higher stillbirth rates in both high income countries and low income countries. In our study, 60% of stillbirth occurred in families with earning <10,000 Pak rupees per month (100 USD) and most of family heads were unemployed. Similar figures of prevalent poverty contributing to high stillbirths have been reported in other studies.^16^,^19^,^21^ In our study, 40% of mothers and 23.36% of fathers did not have formal education at al. Thus, lack of education may be one of the contributing risk for stillbirths.^22^,^23^

## CONCLUSION

Our study showed that stillbirths are more common in primiparous mothers. As such awareness sessions by skilled birth attendants in the community may be helpful to reduce the stillbirth rate in a given context.
